# Assessing probiotic viability in mixed species yogurt using a novel propidium monoazide (PMAxx)-quantitative PCR method

**DOI:** 10.3389/fmicb.2024.1325268

**Published:** 2024-02-08

**Authors:** Tlaleo A. Marole, Thulani Sibanda, Elna M. Buys

**Affiliations:** Department of Consumer and Food Sciences, University of Pretoria, Pretoria, South Africa

**Keywords:** probiotic, viability, quantitative PCR, *tuf* gene, *Bifidobacterium* species, *Lacticaseibacillus rhamnosus*, yogurt cultures, propidium monoazide

## Abstract

Viability is a prerequisite for any therapeutic benefits associated with the ingestion of probiotic bacteria. Current culture-based techniques are inadequate for the enumeration of probiotics in mixed-species food products. This study utilized a quantitative PCR (qPCR) method coupled with propidium monoazide (PMAxx), and novel species-specific *tuf* gene primers to selectively enumerate *Lacticaseibacillus rhamnosus, Bifidobacterium* spp., and yogurt starter cultures in mixed-species probiotic yogurt. The method was optimized for PMAxx concentration and specificity and evaluated for efficiency and applicability. PMAxx-qPCR showed high specificity to the target organisms in mixed-species yogurt, quantifying only viable cells. The linear dynamic ranges were established over five to seven orders of magnitude. The assay was reliable with an efficiency of 91–99%, *R*^2^ values > 0.99, and a good correlation to the plate count method (*r* = 0.882). The results of this study demonstrate the high selectivity, improved lead time, and reliability of PMAxx-qPCR over the culture-dependent method, making it a valuable tool for inline viability verification during processing and improving probiotic quality assurance for processors and consumers.

## 1 Introduction

Fermented dairy products are considered excellent carriers of probiotics due to their consumers' general acceptance as health-promoting foods (Nyanzi et al., 2021; Sakandar and Zhang, 2021). Among the fermented dairy products, yogurt is the most popular and consumed probiotic product, with a market share of around 37% (Sakandar and Zhang, 2021). Probiotics are associated with several health benefits, such as gut microbiota stabilization, antimicrobial activity against pathogens, improved antioxidant activity, and therapeutic effects against allergies, inflammatory bowel diseases, and diarrhea (Roobab et al., 2020; Nyanzi et al., 2021). The recommended minimum dosage required for probiotics to impart therapeutic benefits to the host is 10^6^-10^7^ colony-forming units (CFU) per gram (g) or milliliter (ml) at the time of product consumption (Ranadheera et al., 2017; Fazilah et al., 2018; Yao et al., 2020). This corresponds to 10^8^-10^9^ CFU per 100 g or 100 ml serving. Hence, it is a prerequisite to determine probiotic viability during product manufacturing and storage to ensure that the minimum therapeutic dosage is maintained and consumer's expectations of probiotic quality are met. The current methods of probiotic viability determination are based on standardized culture-based techniques (Davis, 2014; Jackson et al., 2019; Vinderola et al., 2019). However, these methods have many limitations (Davis, 2014; Vinderola et al., 2019), and their use for specific quantification of closely related probiotics and starter cultures in mixed-species fermented dairy products such as yogurt is challenging due to possible similarity in growth conditions and shared biochemical properties (Tabasco et al., 2007). Hence, there is a need for alternative methods that can overcome the limitations of culture-based methods for probiotic quantification. Several culture-independent methods such as flow cytometry, real-time quantitative PCR (qPCR), digital PCR (dPCR), and Next Generation Sequencing (NGS) have been considered as alternatives for probiotic enumeration (Wilkinson, 2018; Jackson et al., 2019; Hansen et al., 2020; Nyanzi et al., 2021). Among these methods, qPCR-based methods are commonly used for microbial quantification in fermented dairy products (García-Cayuela et al., 2009; Scariot et al., 2018; Fan et al., 2021; Yang et al., 2021; Shi et al., 2022). These qPCR-based methods are highly selective, sensitive and have short results turnaround time (Fan et al., 2021; Shehata et al., 2023). Quantitative PCR methods use sequence-specific oligonucleotide probes with fluorophores or fluorescent DNA intercalating dyes for real-time continuous detection and amplification of the target DNA from a food sample (Zhang and Fang, 2006; Davis, 2014; Agrimonti et al., 2019). As the target DNA sequence is amplified during qPCR, fluorescence from the intercalating dye or probes increases. The cycle threshold (*Ct*) values are measured during the exponential phase of the amplification curve when fluorescence has accumulated above the background noise (Davis, 2014). Plotting the *Ct* values against known DNA copies allows for direct determination of probiotic quantity in the sample (Zhang and Fang, 2006). The main challenge with qPCR-based methods is their inability to differentiate between DNA from dead and live cells (Fittipaldi et al., 2012; Huang et al., 2018). This limitation can be solved using viability DNA intercalating dyes (Nocker et al., 2006; Nyanzi et al., 2021). At present, there are three types of viability dyes used to prevent the amplification of DNA from dead cells, namely, ethidium monoazide (EMA), propidium monoazide (PMA, next-generation dye) and PMAxx (new generation dye), an improved version of PMA (Shehata and Newmaster, 2021; Kallastu et al., 2023). These dyes are membrane impermeant DNA intercalating dyes that only penetrate the cell membranes of dead cells (Nocker et al., 2006; Fittipaldi et al., 2012). When exposed to bright light, the azide group of the dye releases a highly reactive nitrene molecule, which forms a covalent crosslink with the DNA of dead cells (Nocker et al., 2006; Fittipaldi et al., 2012). The resulting DNA-dye complex is insoluble and is removed with the cell debris during the DNA isolation process (Nocker and Camper, 2006).

While PMA-qPCR-based methods are regarded as sensitive and reliable in quantifying probiotic viability in foods, the methods currently available focus on quantifying a single probiotic species (Scariot et al., 2018; Shehata and Newmaster, 2021; Shehata et al., 2023). Only a few studies have reported the application of PMA-qPCR in mixed-species probiotic fermented dairy products (García-Cayuela et al., 2009; Yang et al., 2021; Shi et al., 2022). However, none of the studies have reported the use of PMA-qPCR to quantify probiotic *Lacticaseibacillus rhamnosus, Bifidobacterium* spp., and starter cultures in mixed-species probiotic yogurt. Secondly, the application and validation of PMAxx, a new generation viability dye, has never been reported for quantifying mixed species probiotics in yogurt. Hence, the objective of this study was to develop a qPCR method coupled with PMAxx and novel species-specific primers targeting the translation elongation factor EF-TU (*tuf* ) gene with the aim of selectively quantifying viable *L. rhamnosus, Bifidobacterium* spp., and starter cultures in mixed-species probiotic yogurt.

## 2 Materials and methods

### 2.1 Bacterial reference strains and growth conditions

*Streptococcus thermophilus* NCIMB 8510 and *Lactobacillus delbrueckii* subsp. *bulgaricus* NCIMB 11778 were obtained from the NCIMB Ltd., (Aberdeen, Scotland). While *Lacticaseibacillus rhamnosus* ATCC 53103, *Limosilactobacillus fermentum* ATCC 9338, *Lactiplantibacillus plantarum* ATCC 14917, *Bifidobacterium breve* ATCC 15700 and *Bifidobacterium bifidum* ATCC 11863 were obtained from KWIK-STIK™, Microbiologics (MN, USA). The lyophilized bacterial reference strains were cultured twice in 10 ml sterile de Man, Rogosa, and Sharpe (MRS) broth (Neogen, Lansing, MI, USA) for non*-Bifidobacterium* spp. and MRS broth supplemented with 0.05% L-cysteine (Sigma-Aldrich, St. Louis, USA) (MRSc), for *Bifidobacterium* spp. The MRS broth cultures were incubated at 37°C for 24 h under aerobic conditions, while MRSc broth cultures were incubated at 48 h−72 h under anaerobic conditions using anaerobic gas generating sachets (AnaeroGen^TM^ 2.5L, Oxoid Ltd, Basingstoke, UK). Stock cultures in 25% (v/v) sterile glycerol and cryo-beads, were stored at −80°C until needed for use. In addition to reference cultures, *S. thermophilus* and *L. bulgaricus* were also isolated from a commercial yogurt starter culture (LYOFAST Y 259 A, SACCO, Como, Italy). The identity of the isolates from the commercial starter cultures and the reference species used in this study was confirmed using matrix-assisted laser desorption ionization-time of flight (MALDI-ToF) Biotyper (Bruker, Bremen, Germany).

### 2.2 Species-specific primer design

The target gene selection for primer design was based on the multiple comparisons between the *tuf* and 16S rRNA gene sequences retrieved from the NCBI GenBank database.^1^ The gene sequences of typical representative reference strains, namely *B. bifidum* ATCC 29521, *L. rhamnosus* ATCC 53103, *L. delbrueckii* subsp. *bulgaricus* ATCC 11842 and *S. thermophilus* ATCC 19258 were aligned and analyzed using the Multiple Sequence Comparison by Log Expectation (MUSCLE) program.^2^ The alignment results were viewed using the Jalview software (Waterhouse et al., 2009). Species-specific primers (Table 1) for *Bifidobacterium* spp., *L. rhamnosus*, and *L. delbrueckii* were designed using the free online software primer 3 plus.^3^ The primers were designed using the *tuf* gene sequences of *B. bifidum* BCRC 11844 (Accession Number: FJ549340.1), *L. rhamnosus* strain W6 (Accession Number: JN694773.1), and *L. delbrueckii* strain A23 (Accession Number: JN694768.1) retrieved from the NCBI GenBank database. *S. thermophilus-*specific primers were obtained from Fan et al. (2021). The designed primers were synthesized by Integrated DNA Technologies (IDT, Brussels, Belgium).

**Table 1:** Species-specific primers for probiotics and yogurt cultures.

**Organism**	**Primer sequence**		**Primer name**	**Location within a gene**	**References**
*Bifidobacterium* spp.	F	5′-AAGCCGTTCCTGATGCCTATC-3′	Bb-1F	398–418	This study
		R	5′-GAGGTAACGGTGGTGGTCTG-3′	Bb-1R		527–546	
*L. delbrueckii*	F	5′-AGACTCTTGACTTGGGTGAAGC-3′	Ldb-1F	112–133	This study
		R	5′-GTTCTGTGGGTCTTGATTGAGC-3′	Ldb-1R		211–232	
*L. rhamnosus*	F	5′-ATCGATCGTGGTACGGTTAAGG-3′	Lcr-1F	12–33	This study
		R	5′-ACCAAGATCCAAGGTCTTACGG-3′	Lcr-1R		107–128	
*S. thermophilus*	F	5′-CGTGGTGTTGTTCGTGTTAATGA-3′	ST-F		Fan et al. (2021)
		R	5′-CGGCAATACCTTCATCAAGTTGT-3′	ST-R			

F, Forward Primer; R, Reverse Primer.

### 2.3 Primer specificity and PCR conditions

#### 2.3.1 DNA extraction

The total genomic DNA was extracted from the cell pellets obtained from the pure bacterial cultures and yogurt samples using NucleoSpin^®^ Microbial DNA and NucleoSpin^®^ Food DNA isolation kits (Macherey-Nagel Gmbh & Co. KG, Düren, Germany) respectively. DNA concentration was determined using Qubit^TM^ 4 Fluorometer and dsDNA High Sensitivity (HS) working solution (1×) (Invitrogen™, Thermo Fisher Scientific, Waltham, USA). The DNA quality was determined using NanoDrop ND-1000 UV/Vis spectrophotometer V 3.8.1 (Peqlab, Erlangen, Germany) at A260/A280.

#### 2.3.2 Primer specificity verification

The specificities of the designed primers for *L. delbrueckii* subsp. *bulgaricus, L. rhamnosus* and *Bifidobacterium* spp. were checked *in silico* using the Basic Local Alignment Search Tool (BLAST) program from the NCBI website^4^ against the nucleotide collection (nt) and Refseq representative genomes database. Experimental primer specificity verification was performed using DNA isolated from the monocultures and five mixed-species samples (MRS broth) containing an equal concentration of 10^8^ Cells/ml of each reference species. The mixed-species sample compositions were as follows: Sample A: *S. thermophilus, L. subsp. bulgaricus, L. rhamnosus, L. plantarum, L. fermentum, B. bifidum*, and *B. breve* (All species). Samples B to E were the negative controls and contained all the species minus *S. thermophilus, L. delbrueckii* subsp. *bulgaricus, L. rhamnosus*, and *Bifidobacterium* spp., respectively. The DNA was diluted to 5 ng using PCR-grade ultra-pure water prior to the qPCR assay. The non-specific amplification and primer dimers were checked using melt curve analyses and gel electrophoresis as described in the section 2.3.3 (Real-time qPCR conditions).

#### 2.3.3 Real-time qPCR conditions

Quantitative PCR reactions were conducted in duplicate, and each reaction contained 5.0 μL of 2 × TB Green^®^ Advantage^®^ qPCR Premix consisting of TB Green dye, full-length Taq DNA Polymerase, hot-start antibody, dNTPs, and buffer (Takara Bio Inc, Mountain View, CA, USA), 0.2 μL of forward primer (10 μM), 0.2 μL of reverse primer (10 μM), 1.0 μL of template DNA (0.5 ng) and 3.6 μL of nuclease-free water in a final qPCR volume of 10 μL. Each qPCR run included no template control (NTC), and 1.0 μL of nuclease-free water was used as a template. The qPCR assay was performed on the CFX96 Touch Real-Time PCR Detection System (Bio-Rad, Hercules, CA, USA). Quantitative PCR cycle conditions were as follows: initial denaturation at 95°C for 30 s followed by 35 cycles of denaturation at 95°C for 5 s for all species, annealing at 62°C for 20 s (*L. delbrueckii* subsp. *bulgaricus* and *S. thermophilus*) or 62°C for 15 s (*L. rhamnosus*) and extension at 72°C for 6 s (*L. delbrueckii* subsp. *bulgaricus*), 72°C for 15 s (*L. rhamnosus*) and 72°C for 20 s (*S. thermophilus*). Annealing and extension were carried out as a combined step for *Bifidobacterium* spp. at 60°C for 20 s. Each reaction was held at 4°C for 5 s, followed by the melting curve analysis at 45 to 95°C with an increment of 0.5°C. The cycle threshold (Ct) was calculated automatically using a single threshold mode based on the point at which the threshold has crossed the background levels and at which the exponential phase of the qPCR reaction was reached. The qPCR products were electrophoresed with an ethidium bromide (Invitrogen, Carlsbad, USA) stained 3% agarose gel running at 90 V for 60 min in 0.75 × TAE buffer. The gel was analyzed using a gel documentation system (Gel Doc^TM^ EZ imager, Bio-Rad, California, USA).

### 2.4 PMAxx-qPCR

#### 2.4.1 PMAxx treatment

The effective concentration of PMAxx was determined by treating heat-killed *L. rhamnosus* cells with different concentrations of PMAxx ranging from 50, 75 and 100 μM. Overnight cultures of *L. rhamnosus* in MRS broth (1.5 ml) were heat-treated in a water bath at 95°C for 5 min. PMAxx treatment was performed following a method described by Scariot et al. (2018) and PMAxx supplier protocol but with modifications. The heat-killed bacterial cells were centrifuged at 6000 × *g* for 2 min using a microcentrifuge (Ortoalresa, Madrid, Spain), then washed twice with sterile phosphate-buffered saline (PBS), pH 7.3 (Oxoid Ltd, Basingstoke, UK). The cell pellets were resuspended in 400 μl of ultra-pure water, and a PMAxx^TM^ dye (Biotium Inc., Hayward, CA, USA) stock solution (20 mM) was added to give final concentrations of 50, 75, and 100 μM. All the samples were incubated in the dark for 10 min at room temperature and were subjected to mixing every 1 min. The samples were then placed on ice and exposed to a 500 W halogen light source at a distance of 12 cm (Shao et al., 2016) for 15 min to create a covalent link between PMAxx and DNA. The samples were turned frequently to ensure maximum light exposure was achieved. Upon PMAxx treatment, the samples were centrifuged at 6000 × *g* for 10 min. The obtained cell pellets were subjected to DNA extraction and qPCR.

#### 2.4.2 Determination of PMAxx effectiveness and its effect on live cells

The effect of PMAxx at the concentration of 100 μM was tested on all the target bacterial species used in the study. Aliquoted samples containing bacterial cultures (1.5 ml) of each target species were used to determine the effect of PMAxx on live cells. Samples were divided into two groups, namely control and test samples, which contained live untreated cells and live PMAxx-treated cells, respectively. All samples were 10-fold serially diluted in a 7-point dilution and were spread plated (100 μL) on M17-glucose (*S. thermophilus*), MRS (*L. rhamnosus* and *L. bulgaricus*), and MRSc (*Bifidobacterium* spp.) agars.

The effectiveness of PMAxx in inhibiting the amplification of DNA from dead cells was determined by calculating the percentage of dead cell DNA removal based on the equations 1–4 recommended by the dye manufacturer but with slight modifications.


(1) $$\begin{array}{l} \Delta {\text{Ct}}_{\left(\text{dead}\right)}={\text{Ct}}_{\left(\text{dead PMAxx }-\text{ treated}\right)}\\ \;-{\text{Ct}}_{\left(\text{dead untreated}\right)} \end{array}$$

(2) $$\begin{array}{lll} \text{Fold decrease by PMAxx} & = & 2^{\Delta \text{Ct }\left(\text{dead}\right)} \end{array}$$

(3) $$\begin{array}{lll} \%\text{ Dead cell DNA remaining} & = & \frac{100}{\text{Fold decrease by PMAxx}} \end{array}$$

(4) $$\begin{array}{l} \%\text{ Dead cell DNA removed}=100\\ \;-\%\text{ dead cell DNA remaining } \end{array}$$

#### 2.4.3 Determination of linear dynamic range, efficiency, slope, correlation and limit of quantification

The standard curves were created using the genomic DNA isolated from PMAxx-treated pure cultures of *L. delbrueckii subsp. bulgaricus* NCIMB 11778, *S. thermophilus* NCIMB 8510*, L. rhamnosus* ATCC 53103, *B. breve* ATCC 15700, and *B. bifidum* ATCC 11863 on two different days. The genomic DNA was 10-fold serially diluted in PCR-grade ultra-pure water to the final copy number ranging from 10^7^ to 10^0^ per reaction. The linear dynamic range (LDR), efficiency (E), slope (K), and correlation coefficient (*R*^2^) were determined from the standard curves created by plotting Ct values vs. log DNA copy number. The limit of quantification (LOQ) was determined using the standard curves created by plotting the Ct values vs. log CFU/ml. To plot Ct vs. log CFU/ml, the bacterial cultures of the target species were 10-fold serially diluted in a 7-point dilution and spread-plated (100 μl) as previously described. The qPCR amplification efficiencies were determined using the equation 5 (Broeders et al., 2014):


(5) $$\begin{array}{l} \text{E}=100\;\times \;\left(10^{-1/\text{S}}-1\right) \end{array}$$

Where E is the qPCR amplification efficiency, S is the slope obtained from the standard curve.

The DNA copy number was calculated using the equation 6 and the genome of *B. bifidum* ATCC 11863 (2,211,767 bp),^5^ *B. breve* ATCC 15700 (2,275,660 bp),^6^ *L. delbrueckii subsp. bulgaricus* ATCC 11842 (1,864,998 bp), NCBI: txid 390333 (Van De Guchte et al., 2006); *S. thermophilus* ATCC 19258 (2,102,268 bp), GenBank: CP038020 (Cho et al., 2021), and *L. rhamnosus* GG (3,010,111 bp) GenBank: FM179322.1 (Kankainen et al., 2009).


(6) DNA copy number=DNA amount (ng) × Avogadro's constant (6.022  ×  1023)DNA template length (bp)  ×  MW  ×  CF

Where MW is the average molecular weight of double stranded DNA (660 Da) per base pair and CF is the conversion factor (1 × 10^9^).

#### 2.4.4 Comparison of PMAxx-qPCR method to standard plate count method

The target species' viable counts were determined using the plate count method and PMAxx-qPCR. The culture samples of the target species were prepared on two different days (*n* = 10) in MRS broth. The plate count method described in the section 2.4.2 (Determination of PMAxx effectiveness and its effect on live cells) was used to quantify the target species. To perform PMAxx-qPCR, the bacterial cultures were subjected to PMAxx treatment, DNA extraction, and qPCR.

### 2.5 PMAxx-qPCR method application for viability determination in mixed-species probiotic yogurt during storage

Raw cow's milk collected from the University of Pretoria research farm (Pretoria, South Africa) was pasteurized at 85°C for 30 min. The milk was cooled to 40°C, and inoculated with the bacterial cultures at the final concentration of 1.5 × 10^9^ Cells/ml each. Species mixtures used for fermentation were as follows: Yogurt mixture I: *L. rhamnosus* ATCC 53103, *L. delbrueckii* subsp. *bulgaricus* NCIMB 11778, *S. thermophilus* NCIMB 8510, *B. bifidum* ATCC 11863, *L. plantarum* ATCC 14917, and *L. fermentum* ATCC 9338 and yogurt mixture II: Similar to yogurt mixture I, except *B. bifidum* which was replaced with *B. breve*. The mixtures were incubated at 40°C until pH 4.5 was reached, then cooled and kept at 4°C. The yogurt was aliquoted (3 g) into two (Control sample and PMAxx-treated sample) on days 1 and 30 of storage, and its pH was adjusted to 6.5 with 1 M NaOH (García-Cayuela et al., 2009). Casein micelle was dispersed by adding 1 M tri-sodium citrate (3 ml) followed by centrifugation at 10,000 × *g* for 10 min at 4°C (García-Cayuela et al., 2009). Cell pellets were washed with sterile PBS (Yang et al., 2021) and resuspended in 400 μl ultra-pure water or MRS broth before the PMAxx treatment at 100 μM (except for non-treated yogurt). Cells were then subjected to DNA extraction and qPCR. The bacterial count in pure cultures and in the yogurt were calculated as described by Ilha et al. (2016).

### 2.6 Statistical analysis

GraphPad Prism version 9.0 (GraphPad Software, San Diego, CA, USA) was used to analyse data. The *t*-test was used to determine the statistical difference between the viable counts of untreated and PMAxx-treated cells. Simple linear regression and Bland-Altman method of comparison were used to find the correlation between PMAxx-qPCR and plate count methods. All analyses were conducted in duplicates. *P* values < 0.05 were considered statistically significant.

## 3 Results

### 3.1 Similarity comparison of *tuf* and 16S rRNA gene sequences

The gene sequence identity based on the number of matched nucleotide bases between the representative target species was lower in the *tuf* gene compared to the 16S rRNA gene (Supplementary data). The overall identity of *tuf* gene sequences between the species was 69.31%, which accounted for 685 base matches within a gene length of 988 bp. Whereas, the overall gene sequence identity of 16S rRNA was 81.73%, which accounted for 1,098 base matches within a gene length of 1344 bp. In addition, the 16S rRNA gene copy number within the genomes of the target species ranged from 3 to 9 copies (supplementary data). In contrast, the *tuf* gene copy within the five target species was 1.

### 3.2 Primer specificity verification

**Figure 1:**
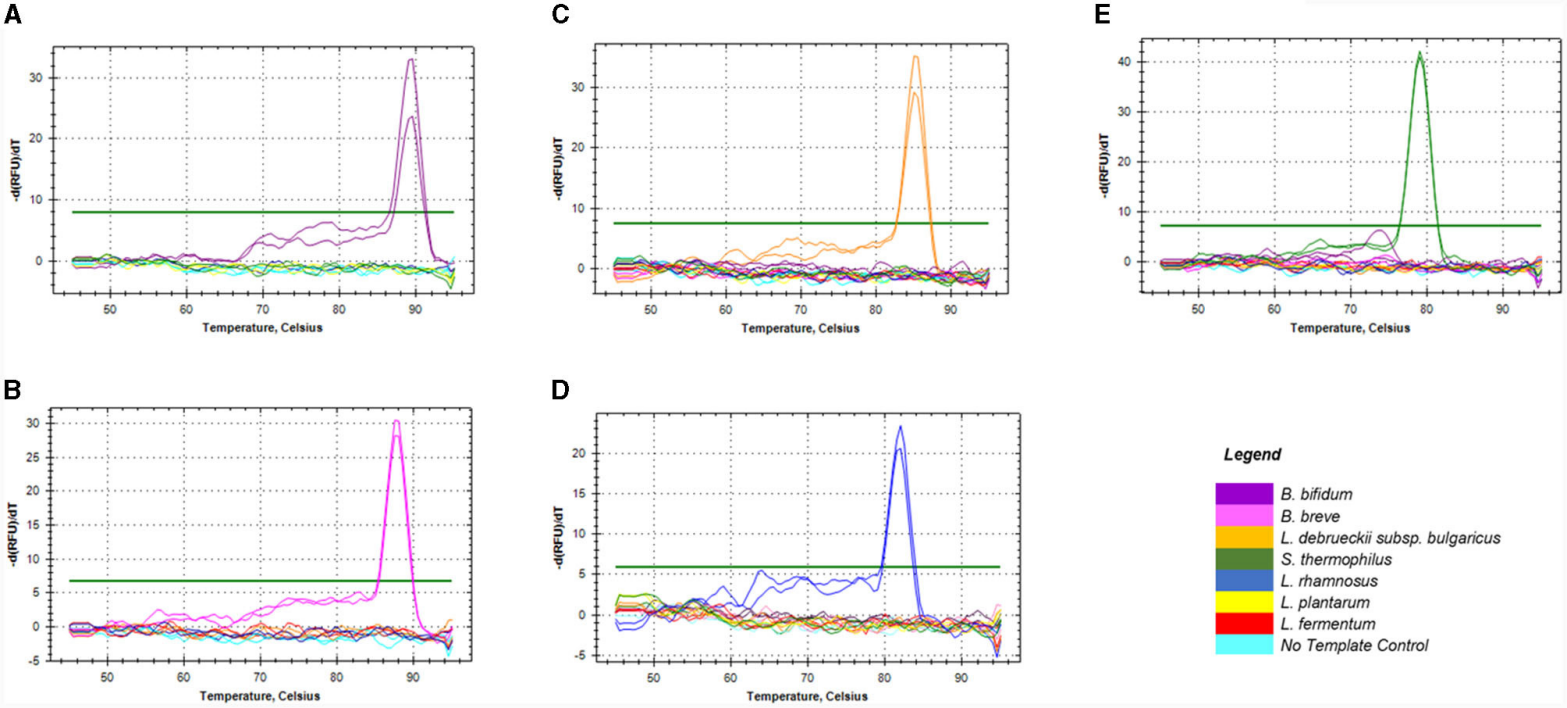
Melt curves showing the specificity of the four sets of primers against DNA of non-target species commonly used in dairy products. Graphs **(A, B)** represented primers for *Bifidobacterium* spp. namely; *B. bifidum* **(A)** & *B. breve* **(B)**, **(C)** for *L. delbrueckii* subsp. bulgaricus, **(D)** for *L. rhamnosus*, and **(E)** for *S. thermophilus*.

The *in silico* specificity verification showed that the designed primers were specific to the target species and could amplify different strains within the same species. Although, *L. rhamnosus* primers could amplify three non-target species during *in silico* PCR, they were still suitable for this study. All the primers used in this study only amplified the target fragment of the bacterial genome during the empirical specificity verification, producing only one melt peak for the target organism in monocultures (Figures 1A–E) and mixed species (Supplementary data). The amplicons with the melt temperatures (Tm) of 89, 87.5, 85, 79, and 82°C corresponding to *B. bifidum* ATCC11863, *B. breve* ATCC 15700, *L. delbrueckii* subsp. *bulgaricus* NCIMB 11778, *S. thermophilus* NCIMB 8510 and *L. rhamnosus* ATCC 53103 were produced, respectively. In addition, single bands with the expected sizes of 149, 121, 118 and 117 bp were produced on gel electrophoresis (data not shown) for *Bifidobacterium* spp., *L. delbrueckii* subsp. *bulgaricus, S. thermophilus*, and *L. rhamnosus*, respectively. There was no formation of artifacts or non-specific products during the qPCR melt curve and gel electrophoresis analyses.

### 3.3 PMAxx concentration optimization and treatment

#### 3.3.1 Effective PMAxx concentration

**Figure 2:**
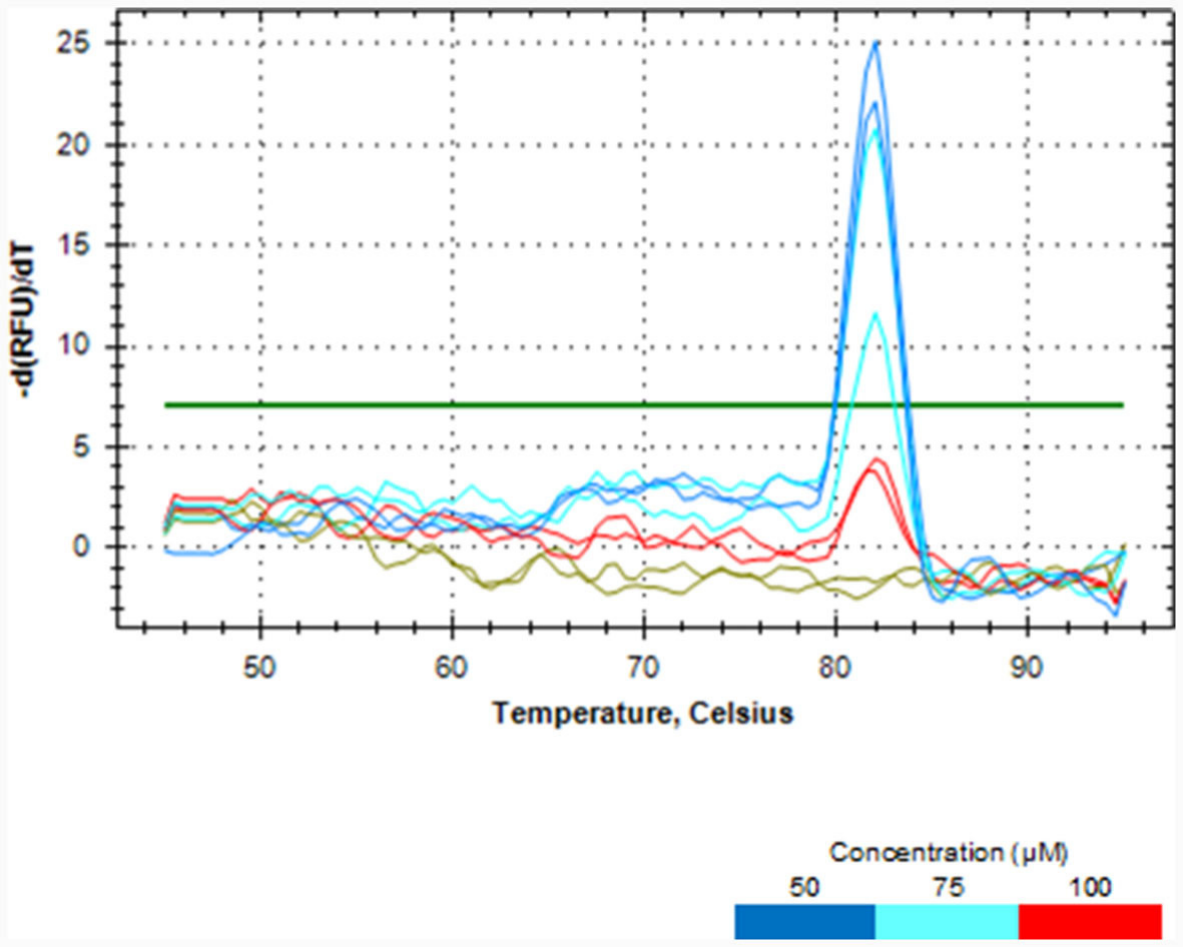
Melt curve analysis showing the effectiveness of different PMAxx concentrations of 50, 75 and 100 μM in removing DNA from dead cells of *L. rhamnosus* ATCC 53103.

Melt curve analysis showed that PMAxx, at a concentration of 100 μM, completely removed DNA from the dead cells of *L. rhamnosus* as no qPCR product was produced, as depicted in Figure 2. PCR amplicons were produced at concentrations of 50 and 75 μM. Hence, the 100 μM PMAxx concentration was chosen as the working concentration for this study.

#### 3.3.2 Effectiveness of PMAxx concentration (100 μM) and its effect on live cells

**Figure 3:**
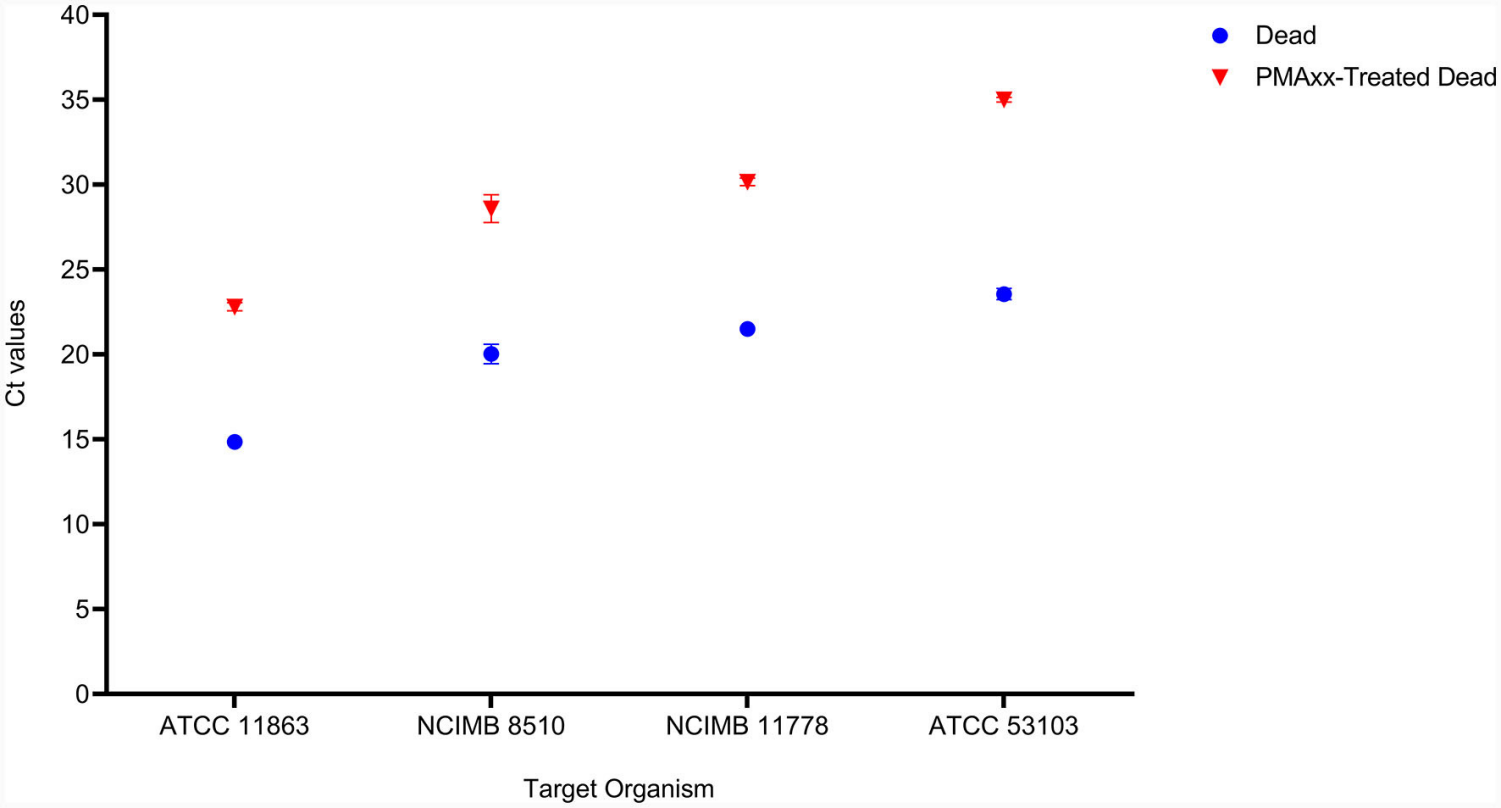
The effectiveness of PMAxx at the concentration of 100 μM on the removal of DNA from dead cells of *B. bifidum* (ATCC 11863), *S. thermophilus* (NCIMB 8510), *L. delbrueckii* subsp. *bulgaricus* (NCIMB 11778), and *L. rhamnosus* (ATCC 53103). Blue points – DNA from dead-untreated cells, and Red points – DNA from dead PMAxx-treated cells.

The effectiveness of PMAxx was affected by the type of media or solution used for treatment. *S. thermophilus* NCIMB 8510 and *B. bifidum* ATCC 11863 cells were observed to be sensitive (viability declined, data not shown) to PMAxx when treated in ultra-pure water (ddH_2_O) at 100 μM. On the contrary, when the two species were treated in MRS broth, PMAxx did not affect their viability. The effectiveness of PMAxx in removing DNA from dead cells was reduced when *L. delbrueckii* subsp. *bulgaricus* cells were treated in MRS broth (data not shown). The Ct values of dead untreated cells were 14.86 ± 0.44, 23.56 ± 0.32, 20.04 ± 0.57, and 20.62 ± 0.14 for *B. bifidum, L. rhamnosus, S. thermophilus*, and *L. delbrueckii* subsp. *bulgaricus*, respectively (Figure 3). The treatment of dead cells with PMAxx at 100 μM resulted in a significant shift in Ct values to 22.81 ± 0.24, 35.01 ± 0.14, 28.59 ± 0.82, and 28.89 ± 0.01, respectively. Hence resulting in a delta Ct >7 for all four species. In general, PMAxx at 100 μM effectively removed 99.6, 100.0, 99.7, and 99.6% (Table 2) of DNA from the dead cells of *B. bifidum* ATCC 11863, *L. rhamnosus* ATCC 53103, *L. delbrueckii* subsp. *bulgaricus* NCIMB 11778 and *S. thermophilus* NCIMB 8510, respectively.

**Table 2:** Removal (%) of dead cells DNA at PMAxx ^TM^ concentration of 100 μM.

**Species**	**Removed dead cells DNA (%)**
*S. thermophilus* NCIMB 8510	99.6
*L. delbrueckii subsp. bulgaricus* NCIMB 11778	99.7
*B. bifidum* ATCC 11863	99.6
*L. rhamnosus* ATCC 53103/GG	100.0

**Figure 4:**
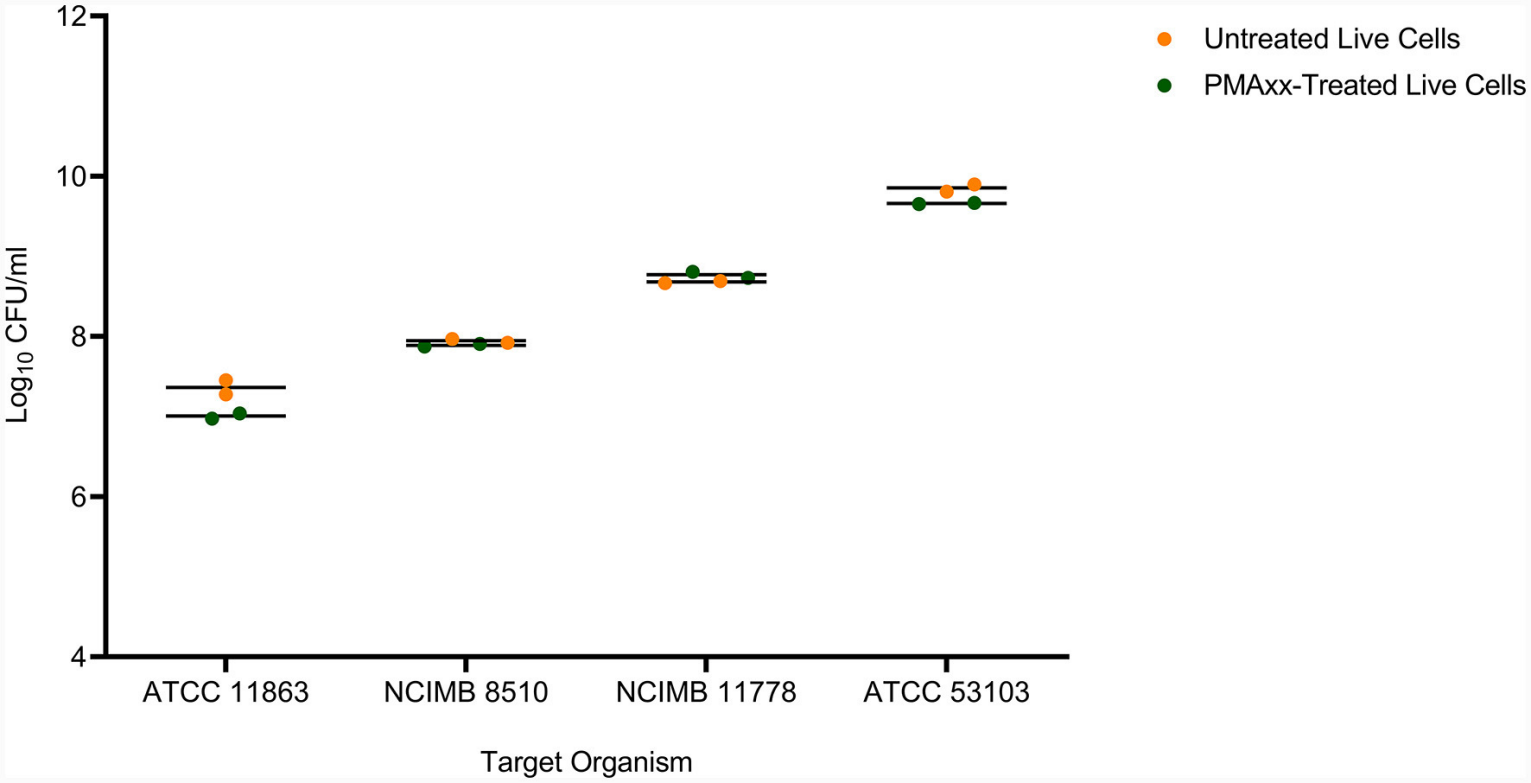
The effect of PMAxx at the concentration of 100 μM on live cells of *B. bifidum* (ATCC 11863), *S. thermophilus* (NCIMB 8510), *L. delbrueckii* subsp. *bulgaricus* (NCIMB 11778), and *L. rhamnosus* (ATCC 53103).

In addition, PMAxx at 100 μM did not affect the viability of live cells of the target LAB species (Figure 4). There was no significant difference (*p* > 0.05) between the viable counts of untreated live and PMAxx-treated live cells for all the target species.

### 3.4 Standard curves: linear dynamic range, efficiency, slope, and correlation determination

**Figure 5:**
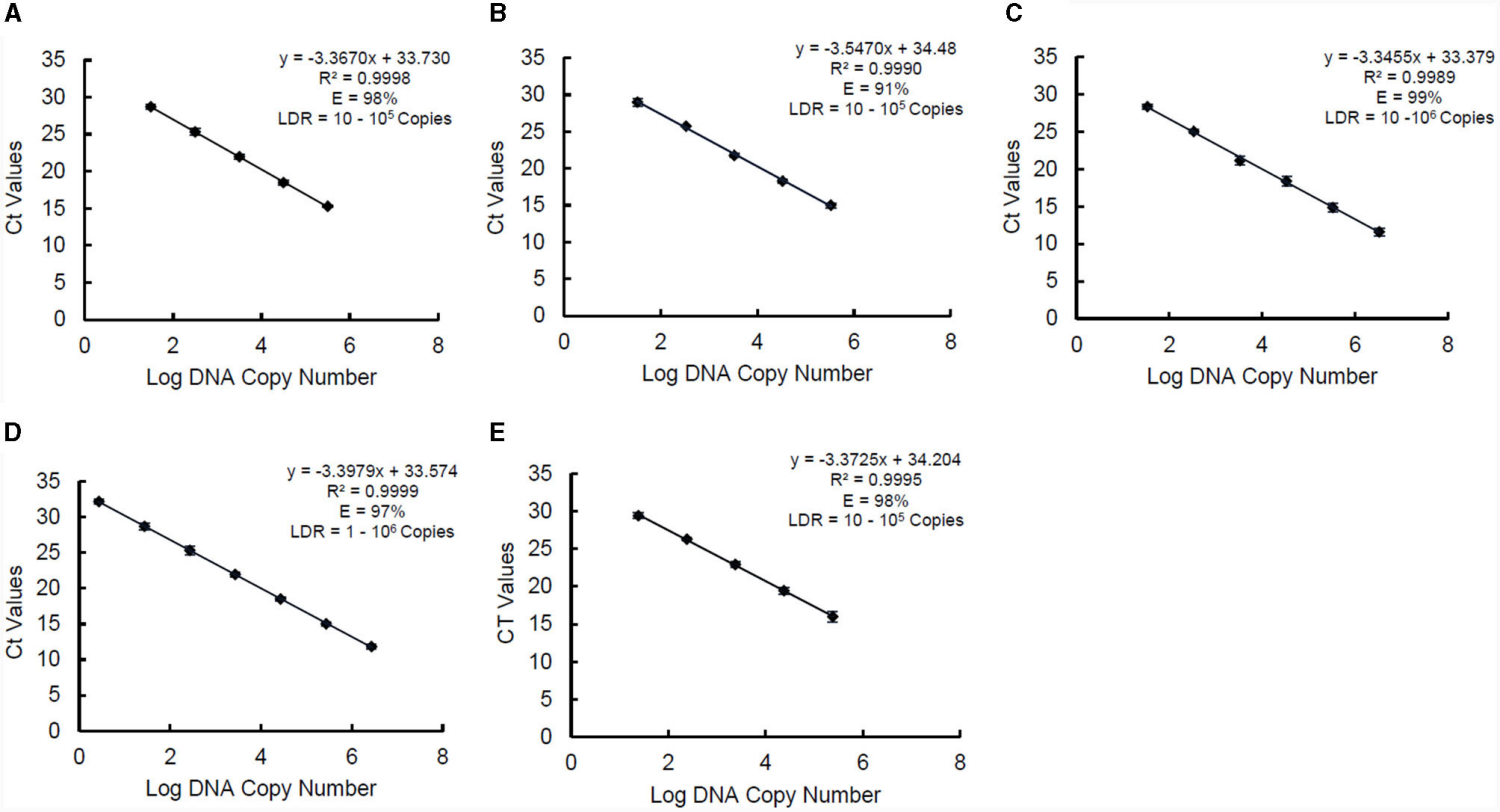
Standard curves of PMAxx-qPCR assay created and used for determining linear dynamic range (LDR), efficiency (E), and slope (K) for *B. bifidum* ATCC 11863 **(A)**, *B. breve* ATCC 15700 **(B)**, *L. delbrueckii* subsp. *bulgaricus* **(C)**, *S. thermophilus* **(D)**, and *L. rhamnosus* **(E)**. Each point represents the mean ± standard deviation of CT values of two independent runs. Each run was carried out in duplicates (*n* = 4).

The standard curve parameters, namely slope (K), efficiency (E), and correlation coefficient (*R*^2^) of two independent qPCR runs for the five target species, are summarized in Table 3. The overall mean of the Ct values and DNA copy numbers obtained from the two independent qPCR runs were used to establish the standard curve parameters for this study (Figure 5). There was a good linear fit (*R*^2^ > 0.99, *p* < 0.0001) between the Ct values and log DNA copy number for all the target species. The replicate test for lack of fit showed that the linear model for all the five species adequately fits the data (*p* > 0.05). The PMAxx-qPCR assays for the five species were efficient (E = 91%−99%) with a slope ranging from −3.55 to −3.35. The linear dynamic ranges (LDR) were determined between 10 and 10^5^ genome copies for *Bifidobacterium* spp. and *L. rhamnosus*, 10 and 10^6^ genome copies for *L*. *delbrueckii* subsp. *bulgaricus*, and 1 and 10^6^ genome copies for *S. thermophilus*. The LOQ was 10^2^ CFU/ml for *B. bifidum* and *S. thermophilus*, 10^3^ CFU/ml for *B. breve* and *L. delbrueckii* subsp. *bulgaricus*, and 10^4^ CFU/ml for *L. rhamnosus* (Supplementary data).

**Table 3:** Quantitative PCR efficiency, slope, correlation coefficient obtained by plotting Ct values against log DNA copy number.

**Species**	**qPCR efficiency (E)**	**Slope (K)**	**Correlation coefficient (R^2^)**
*S. thermophilus* NCIMB 8510	97%	3.3975	0.9997
*L. delbrueckii subsp. bulgaricus* NCIMB 11778	99%	3.3455	0.9963
*B. bifidum* ATCC 11863	98%	3.3670	0.9995
*B. breve ATCC 15700*	92%	3.5470	0.9983
*L. rhamnosus* ATCC 53103/GG	95%	3.4623	0.9980

Data represent the mean values (*n* = 4) of two independent qPCR assays and DNA extractions.

### 3.5 Comparison of PMAxx-qPCR method to standardized plate count method

**Figure 6:**
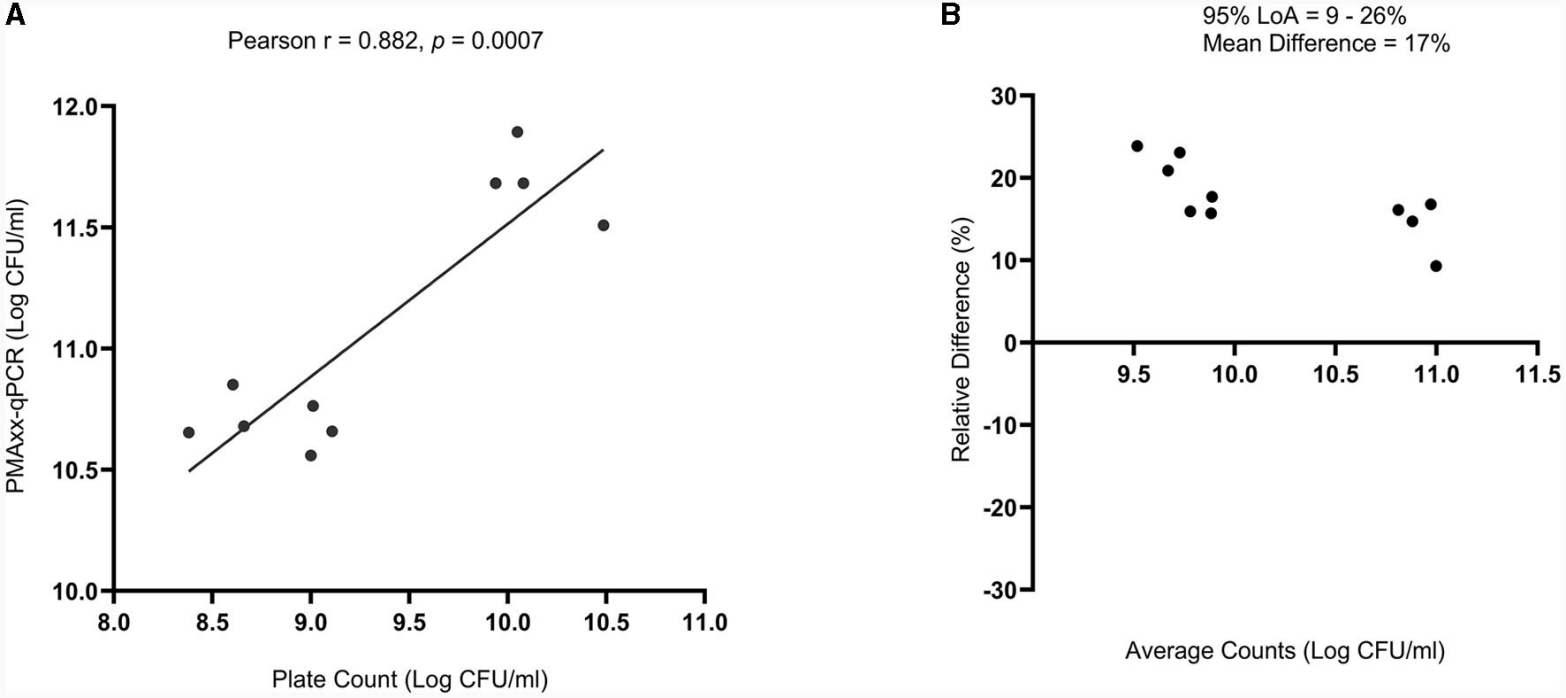
Comparison of PMAxx-qPCR method with standardized plate count method (*n* = 10) using **(A)** simple linear regression and **(B)** Bland-Altman method of comparison (%Difference vs. Average). The Bland-Altman comparison results are expressed as a percentage relative difference [100 × (PMAxx-qPCR count – Plate count)/average] vs. average. LoA means Limits of Agreement.

There was a high correlation between the viable counts of PMAxx-qPCR and the plate count method [Pearson correlation coefficient (*r*) = 0.882 and *p* = 0.0007], Figure 6A. The *p*-value showed that the true value of the coefficient (0.6303) was significantly different from zero. Hence, confirmed a relationship between the two methods. PMAxx-qPCR counts were generally significantly higher (*p* < 0.0001, two-tailed paired *t*-test) compared to plate count with a relative difference of 17% (range: 9%−26%) (Figure 6B, Bland-Altman method of comparison).

### 3.6 Applicability of PMAxx-qPCR method in mixed-species probiotic yogurt

**Figure 7:**
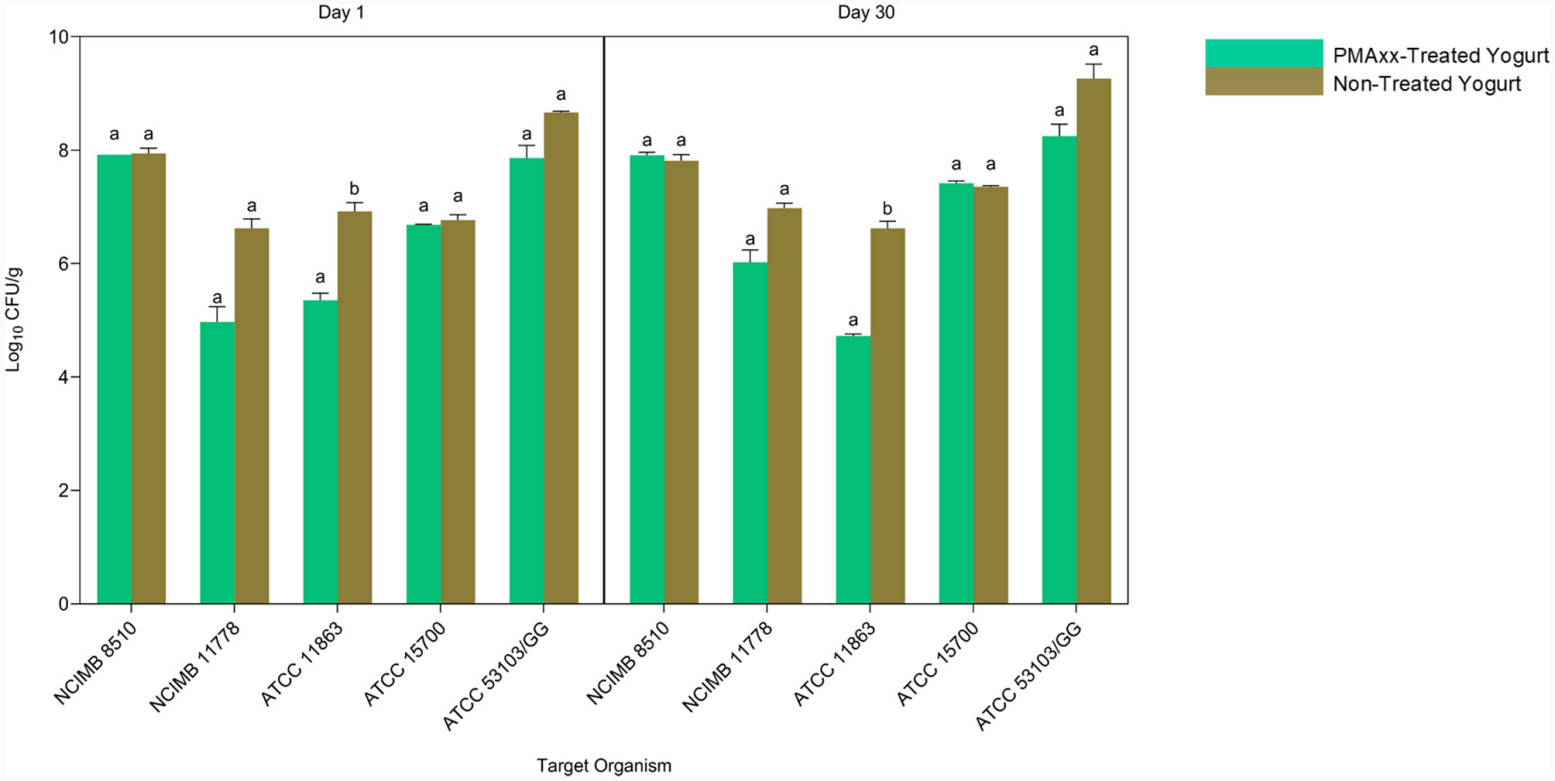
The Applicability of PMAxx-qPCR method in mixed-species yogurt during storage (day 1 and 30). Each bar represents the mean ± SD of duplicate qPCR reactions. DNA extractions from yogurt samples were done in duplicates. Mean values with different letters (a, b) were significantly different (*p* < 0.05).

Quantitative PCR without PMA quantifies all the genomic DNA from live and dead cells. Comparing the qPCR counts of PMAxx-treated and non-treated yogurt, therefore, gives information on the ability of the designed method to quantify viable cells in mixed-species yogurt during storage selectively (Figure 7). *S. thermophilus* NCIMB 8510 counts in PMAxx-treated and non-treated yogurts throughout storage were comparable (*p* > 0.05). This showed that only viable *S. thermophilus* cells were in the yogurt during storage. The qPCR counts for *L. delbrueckii* subsp. *bulgaricus* NCIMB 11778 in PMAxx-treated yogurt were lower than in non-treated yogurt counts (*p* > 0.05) by 1.66 and 0.96 log CFU/ml on days 1 and 30, respectively. Similarly, there was a difference (*p* > 0.05) of 0.80 log CFU/ml on day 1 and 1.02 log CFU/ml on day 30 in *L. rhamnosus* ATCC 53103 counts between PMAxx-treated and non-treated yogurts. The developed PMAxx-qPCR method showed that *Bifidobacterium* spp. have different survival abilities in mixed-species yogurt during storage. There was a significant reduction (*p* < 0.05) in *B. bifidum* ATCC 11863 cell viability on days 1 and 30 by 1.57 and 1.90 log CFU/ml in PMAxx-treated yogurt, respectively. In contrast, *B. breve* ATCC 15700 exhibited better survival ability than *B. bifidum* ATCC 11863. *B. breve* counts in PMAxx-treated and non-treated yogurts were comparable (*p* > 0.05) with no observable difference during storage. In general, qPCR without PMAxx overestimated cell counts by 13% on day 1 and 12% on day 30 (Bland-Altman method of comparison) between the target species.

## 4 Discussion

In our study, species-specific *tuf* gene primers were successfully designed or selected and validated for the selective quantification of *Bifidobacterium* spp*., L. rhamnosus*, and yogurt starter cultures in mixed-species yogurt. In qPCR-based methods, the 16S rRNA gene is commonly used as the target gene for quantifying mixed-species probiotics (García-Cayuela et al., 2009; Yang et al., 2021). However, the copy number of this gene varies between the genomes of LAB probiotic species (Lee et al., 2008; Fan et al., 2021). In addition, the resolution and discriminatory power of the 16S rRNA gene is low compared to that of protein-encoding genes such as *pheS* gene encoding the phenylalanine tRNA ligase subunit alpha, *hsp60* gene encoding the 60-kDa heat shock protein, and *tuf* gene encoding the elongation factor Tu (Yu et al., 2012). Hence, the *tuf* gene, which has high interspecific sequence difference, occurs as a single copy within the bacterial genome (Fan et al., 2021) and evolves at a faster rate than the 16S rRNA gene (Valiunas et al., 2019), was chosen as the target gene in this study.

Ideally, to ensure reliable quantification, qPCR primers should not exhibit sequence homology to the nucleotide sequences of non-target species in the *in silico* and empirical specificity evaluation. In our study, sequence homology was found between the *L. rhamnosus* primers and the sequences of *Lacticaseibacillus* spp., *Lactococcus lactis, Schleiferilactobacillus harbinensis*, and *Lactobacillus coryniformis*. The latter two species are not commonly used as starter cultures or probiotics in the production of yogurt. *S. harbinensis* is associated with non-dairy food products such as fermented cereals, tomato pomace, and spoiled soft drinks (Zheng et al., 2020). It was first isolated from the Chinese fermented vegetable “Suan cai” (Miyamoto et al., 2005). Similarly, *L. coryniformis* is commonly found in fermented vegetable products (Martín et al., 2005). Barring very poor manufacturing practices, the presence of these species in yogurt is unexpected. Similarly, *L. lactis* is primarily used in cheese, buttermilk and sour cream production (Cavanagh et al., 2015; Laroute et al., 2017; Fusieger et al., 2020). Its unintended presence in yogurt is unexpected. Despite the ability of *L. rhamnosus* primers to detect *L. lactis*, the specificity of all the primers designed in this study was generally acceptable. Hence, the primers were suitable for this study as they did not match genome sequences or amplify DNA fragments of commonly used starter cultures and probiotic species in yogurt. It is worth noting that, the use of *L. rhamnosus* primers designed in this study limits the application of this method to the species and combinations used for validation. Comparative genomics can be considered in future studies to identify unique genetic markers and design subspecies and strain-specific primers (Hyeon-Be et al., 2020; Lee et al., 2022). This will enable broad application of PMAxx-qPCR methods for quantifying probiotics in different mixed-strain yogurt products.

Previous studies have shown that the PMA-DNA complex formation is dependent on the PMA concentration. For example, Shao et al. (2016), Shehata and Newmaster (2021), and Shehata et al. (2023) showed that qPCR signal from dead cells is reduced with increasing PMA concentration. These studies showed that a saturation point could be reached, resulting in no further effect if PMA concentration is increased beyond the optimum. Hence, finding an optimum PMA concentration to inhibit qPCR signal from dead cells effectively is essential. In our study, 50 μM was chosen as the starting concentration during PMAxx optimization as it was previously reported to be effective on other probiotic species (Scariot et al., 2018; Shehata and Newmaster, 2021; Shehata et al., 2023). However, in this study, 100 μM was an optimum concentration that effectively removed DNA from dead cells. In agreement with our findings, a recent study showed that PMAxx at high concentration completely removed DNA from high counts of dead cells of *Salmonella* Enteritidis (Thilakarathna et al., 2022). The PMAxx-DNA cross-linkage can be affected by different factors such as conditions of light exposure (light source, time, distance), bacterial species, the target gene (Shao et al., 2016), killing treatment (Yang et al., 2021), sample pH and turbidity (Fittipaldi et al., 2012). Since these factors are inconsistent in PMA-qPCR methods, they may have contributed to the difference in optimum PMA concentrations between this study and the literature.

At higher concentrations, PMAxx tends to adversely affect the counts of live cells (Thilakarathna et al., 2022). We observed a similar effect of PMAxx on live cells of *B. bifidum* and *S. thermophilus* when treated in a transparent medium (ultra-pure water). Hence, to overcome this, *Bifidobacterium* spp. and *S. thermophilus* were treated in MRS broth, while *L. rhamnosus* and *L. delbrueckii* subsp. *bulgaricus* were treated in ultra-pure water. Thilakarathna et al. (2022) attributed the ability of PMAxx to affect live cells at low counts to possible inactivation post-photoactivation step, thus allowing active PMAxx to be carried over to the lysis tube where it can form a crosslink with DNA from live cells post lysis. In addition, PMAxx treatment possibly modifies the surface charge (to less negative) of live cells with compromised cell membranes, enabling their attachment to the polypropylene tube wall (negatively charged) (Thilakarathna et al., 2022). Hence, the transfer of cells to the next tube in the subsequent step leaves the attached cells behind, resulting in a loss of viable cell counts (Thilakarathna et al., 2022).

Ideally, the qPCR assay should have an efficiency of 100%, signifying a doubling of the DNA template per cycle (Svec et al., 2015). However, practically, this is rare to achieve (Svec et al., 2015). Hence, the efficiency of a suitable qPCR method should be 90%−110% (Broeders et al., 2014). Factors such as target sequence and designed primers (primer dimers and hairpin formation) may lead to low qPCR efficiency (Svec et al., 2015; Langlois et al., 2021). The efficiency of the PMAxx-qPCR method designed in this study was within the generally acceptable range. This shows that the primers used in this study were efficient, and the assay is reliable for quantifying probiotics and starter cultures. In general, the qPCR assay was highly sensitive. However, the LOQ for *L. rhamnosus* was high. Notwithstanding this constraint, the method is still suitable for probiotic quality control, given that the LOQ falls below the minimum probiotic standard or therapeutic levels. The high sensitivity of this protocol makes it suitable for quantifying target species appearing in low amounts in complex and mixed species products (Shehata et al., 2023), such as yogurt.

Furthermore, our findings show that the PMAxx-qPCR method can be used as a predictor of standardized plate counts, as indicated by a high Pearson correlation coefficient. Other studies have reported similar findings (Hansen et al., 2020; Shehata and Newmaster, 2021; Shehata et al., 2023). The discrepancy between the viable counts of the two methods, favoring qPCR assay, aligns with the findings of a previous study (Hansen et al., 2020). This can be attributed to the high counts of the PMAxx-qPCR method due to its ability to detect and quantify viable but non-culturable (VBNC) cells (Kibbee and Örmeci, 2017; Liu et al., 2018). Plate count methods cannot detect cells in the VBNC state (Shao et al., 2016; Jackson et al., 2019; Shehata et al., 2023). Cells in this state are still viable and metabolically active but have lost their culturability (Jackson et al., 2019; Hu et al., 2022). Several harsh conditions, such as fermentation, cryopreservation, lyophilisation, and storage, can induce a VBNC state as a protective mechanism in probiotics (Davis, 2014; Jackson et al., 2019). The underestimation of viable counts by plate count method can lead to inadvertent rejections of probiotic products whose counts fall below the minimum standard levels when, in fact, the actual number of viable cells in the product could be higher due to VBNC cells that remain uncounted. This will have cost implications for probiotic manufacturers. On the contrary, the ability of the PMAxx-qPCR method to detect VBNC cells will improve probiotic quality assurance and efficacy for processors and consumers. Compared to the plate count method, this PMAxx-qPCR assay is rapid, with quantification results obtained within a few hours (~8 h). However, further optimisation of this assay for simultaneous detection and amplification of all target species is possible and will reduce results turnaround time significantly.

Ideally, the use of standard curves obtained from the food matrix inoculated with the target species is recommended for a reliable quantification of viable cells in a food product (Postollec et al., 2011). However, as was done in this study, standard curves constructed from pure cultures give a measure of the efficiency of the qPCR reactions.

Probiotic viability determination in yogurt throughout storage is crucial for adherence to regulatory requirements and probiotic quality assurance in the dairy industry. The PMAxx-qPCR method developed in this study can selectively quantify viable cells of probiotics and starter cultures in mixed-species yogurt during storage. The relative viability loss of the five target species during yogurt storage, as indicated by higher counts of qPCR than PMAxx-qPCR, was in agreement with other studies (Scariot et al., 2018; Shi et al., 2022). The designed PMAxx-qPCR assay further showed that while some probiotics and starter cultures can fully maintain viability during processing and storage, other lost viability at different rates. This shows that some species are susceptible to yogurt processing stress while others are resilient. The findings of this study demonstrate the selectivity, sensitivity, and reliability of this PMAxx-qPCR method, which can detect VBNC cells and viability loss in mixed-species yogurt during storage.

## 5 Conclusion

This study outlines a real-time qPCR protocol for viability enumeration of probiotics in mixed species yogurt. The method which is based on newly developed species-specific primers for *L. rhamnosus, Bifidobacterium* spp. and *L. delbrueckii* subsp. *bulgaricus* and an optimized PMAxx-qPCR reaction protocol has a high sensitivity and is reliable. Moreover, the method has very high correlation with the standard viability plate counts, albeit with a consistently higher prediction rate, presumably due to its ability to enumerate cells in the VBNC state. With such a high sensitivity and short turnaround time, the qPCR protocol will be a good proposition for the inline viability quality assurance in probiotic yogurt processing. However, it must be emphasized that the protocol is applicable only to yogurt incorporated with probiotic species and yogurt starter cultures used in this study. Hence, products containing different species of probiotics would require optimization. Moreover, it would be necessary to do a detailed cost comparison analysis with other available methods of viability quantification, considering that industries could be at different levels of capitalization.

## Data availability statement

The original contributions presented in the study are included in the article/Supplementary material, further inquiries can be directed to the corresponding author.

## Author contributions

TM: Conceptualization, Data curation, Formal analysis, Investigation, Methodology, Software, Validation, Visualization, Writing – original draft. TS: Conceptualization, Methodology, Supervision, Writing – review & editing. EB: Conceptualization, Funding acquisition, Methodology, Resources, Supervision, Writing – review & editing.

## Footnotes

^1^ https://www.ncbi.nlm.nih.gov/

^2^ https://www.ebi.ac.uk/Tools/msa/muscle/

^3^ https://www.primer3plus.com/

^4^ https://www.ncbi.nlm.nih.gov/tools/primer-blast/

^5^ https://genomes.atcc.org/genomes/0900f1128a5a4e56

^6^ https://genomes.atcc.org/genomes/efcf0d5d0df5440a
