# Supplementary material for: Assessing probiotic viability in mixed species yogurt using a novel propidium monoazide (PMAxx)-quantitative PCR method
Source: Front Microbiol. 2024 Feb 8;15:1325268. doi: 10.3389/fmicb.2024.1325268 (PMC10882272; doi:10.3389/fmicb.2024.1325268)
Supplement: Supplementary file 1 [file Data_Sheet_1.pdf]

Supplementary Data

Table S1. Gene copy number of 16s rRNA and tuf genes for the five target reference species.

Data Source	Organism	Strain Number	Copies	
			16S rRNA	*tuf*
ATCC website (genomes.atcc.org)	*B. bifidum*	ATCC 11863	3	1
ATCC website (genomes.atcc.org)	*B. breve*	ATCC 15700	3	1
NCBI, Genbank CR954253.1	*L. delbrueckii subsp. bulgaricus*	ATCC 11842	9	1
ATCC website (genomes.atcc.org)	*S. thermophilus*	ATCC 19258	5	1
NCBI, Genbank FM179322.1	*L. rhamnosus*	ATCC 53103	5	1

Table S2. Identity Matrix (%) of tuf gene sequences

Organism	Strain	Identity (%)				Overall Identity (%)	Overall Variation (%)
		ATCC 29521	ATCC 19258	ATCC 11842	ATCC 53103		
Bifidobacterium bifidum	ATCC 29521	100.00	63.83	66.29	68.24	69.31	30.7
Streptococcus thermophilus	ATCC 19258	63.83	100.00	71.31	71.26		
Lactobacillus delbrueckii subsp. bulgaricus	ATCC 11842	66.29	71.31	100.00	74.90		
Lacticaseibacillus rhamnosus	ATCC 53103	68.24	71.26	74.90	100.00		

Analysed using Multiple Sequence Comparison by Log Expectation (MUSCLE) program (https://www.ebi.ac.uk/Tools/msa/muscle/).

Table S3. Identity Matrix (%) of 16S rRNA gene sequences

Organism	Strain	Identity (%)				Overall Identity (%)	Overall Variation (%)
		ATCC 29521	ATCC 19258	ATCC 11842	ATCC 53103		
Bifidobacterium bifidum	ATCC 29521	100.00	77.20	78.28	79.16		
Streptococcus thermophilus	ATCC 19258	77.20	100.00	83.37	84.62	81.73	18.27
Lactobacillus delbrueckii subsp. bulgaricus	ATCC 11842	78.28	83.37	100.00	87.73		
Lacticaseibacillus rhamnosus	ATCC 53103	79.16	84.62	87.73	100.00		

Analysed using Multiple Sequence Comparison by Log Expectation (MUSCLE) program (https://www.ebi.ac.uk/Tools/msa/muscle/).

Table S4. Real-Time qPCR Primer Properties

Primer Name	Primer Length (bp)	Amplicon Size (bp)	Hairpin 3' end (Kcal/mol)	Primer Tm (°C)	GC (%)	Self complementarity	Self 3' Complementarity
ST-F	23	118	1.46	60.00	43.48	4.00	2.00
ST-R	23		-0.50	59.56	43.48	6.00	6.00
Bb-1F	21	149	-0.27	60.20	52.38	3.00	3.00
Bb-1R	20		-0.27	60.04	60.00	3.00	1.00
Ldb-1F	22	121	-1.26	60.22	50.00	5.00	2.00
Ldb-1R	22		0.97	59.51	50.00	2.00	2.00
Lcr-1F	22	117	0.97	59.97	50.00	6.00	0.00
Lcr-1R	22		0.97	59.76	50.00	6.00	1.00

Legend: Tm – Melt Temperature

```
  1  CGTGGACTGC  CCGGGCCACG  CCGACTTCGT  GAAGAACATG  ATCACCGGTG
 51  CTGCCCAGAT  GGATGGCGCC  ATCCTCGTTG  TGGCCGCCAC  CGACGGCCCG
101  ATGGCCCAGA  CCCGCGAGCA  CGTTCTGCTC  GCCCGTCAGG  TGGGTGTCCC
151  GCGTATCCTC  GTCGCCCTGA  ACAAGTGCGA  CATGGTCGAG  GACGAGGAGC
201  TCATCGAGCT  CGTTGAGGAA  GAGGTCCGTG  ACCTCCTCGA  CGAGAACGGC
251  TTCGACCGCG  ACTGCCCGGT  CATCCGTACC  TCCGCCTACG  GCGCTCTGCA
301  CGATGACGCT  CCGGACCACG  ACAAGTGGGT  CCAGACCGTC  AAGGACCTGA
351  TGGACGCCGT  CGACGAGTAC  ATCCCGACCC  CGGTTCACGA  CCTGGACAAG
401  CCGTTCCTGA  TGCCTATCGA  GGACGTCTTC  ACCATCTCCG  GCCGTGGTAC
451  CGTCGTCACC  GGTCGTGTTG  AGCGTGGCCA  GCTGGCCGTC  AACACCCCGG
501  TCGAGATCGT  CGGCATCCGC  CCGACCCAGA  CCACCACCGT  TACCTCCATC
551  GAGACGTTCC  ACAAGACCAT  GGACGCCTGC  GAGGCTGGCG  ACAACACCGG
601  TCTGCTGCTC  CGCGGCATCA  ACCGTACGGA  CGTCGAGCGT  GGTCAGGTTG
651  TGGCCAAGCC  GGGCTCCGTC  ACCCCGCACA  CCAAGTTCGA  GGGCGAAGTC
701  TACGTGCTGA  CCAAGACGAG  GGCGGCCGTC  ACTCGCCGTT  CTTCTCCAAC
751  TACCGTCCGC  AGTCTACT
```

B

```
  1  CTTCAGGCCG  TATCGACCGT  GGTACTGTTA  AGGTTGGCGA  CAGCGTTGAA
 51  ATCGTTGGTT  TGGTAGAAAA  GGTCTTGACT  TCAGTTGTAA  CTGGCTTGGA
101  AATGTTCCAC  AAGACTCTTG  ACTTGGGTGA  AGCCGGCGAC  AACGTTGGTG
151  TATTGCTCCG  TGGTGTCGAC  CGTGACCAAA  TCGTTCGTGG  CCAAGTTTTG
201  GCAGCTCCAG  GCTCAATCAA  GACCCACAGA  ACTTTCAAGG  GTCAAGTCTA
251  CATCTTGAGT  AAGGAAGAAG  GT
```

C

```
  1  CTTCAGGTCG  TATCGATCGT  GGTACGGTTA  AGGTCGGCGA  TGAAGTTGAA
 51  ATCATCGGTT  TGAAGCCAGA  TGTTCTCAAG  TCCACCGTTA  CCGGTCTTGA
101  AATGTTCCGT  AAGACCTTGG  ATCTTGGTGA  AGCCGGCGAT  AACGTTGGTG
151  TCCTGCTTCG  TGGGATCAAC  CGTGACCAAG  TTGAACGTGG  CCAAGTTTTG
201  GCAAAGCCAG  GTTCAATCCA  ATTGCACAAC  AAGTTCAAGG  GTGAAGTTTA
251  TATCTTGACA  AAAGAAGAAG  GT
```

Figure S1. Designed primer sets (Yellow-Forward Primer & Purple-Reverse Primer) flanking target regions on the tuf genes of B. bifidum (A), L. delbrueckii (B) and L. rhamnosus (C). Primers were designed using the online software primer 3 plus (https://www.primer3plus.com/).

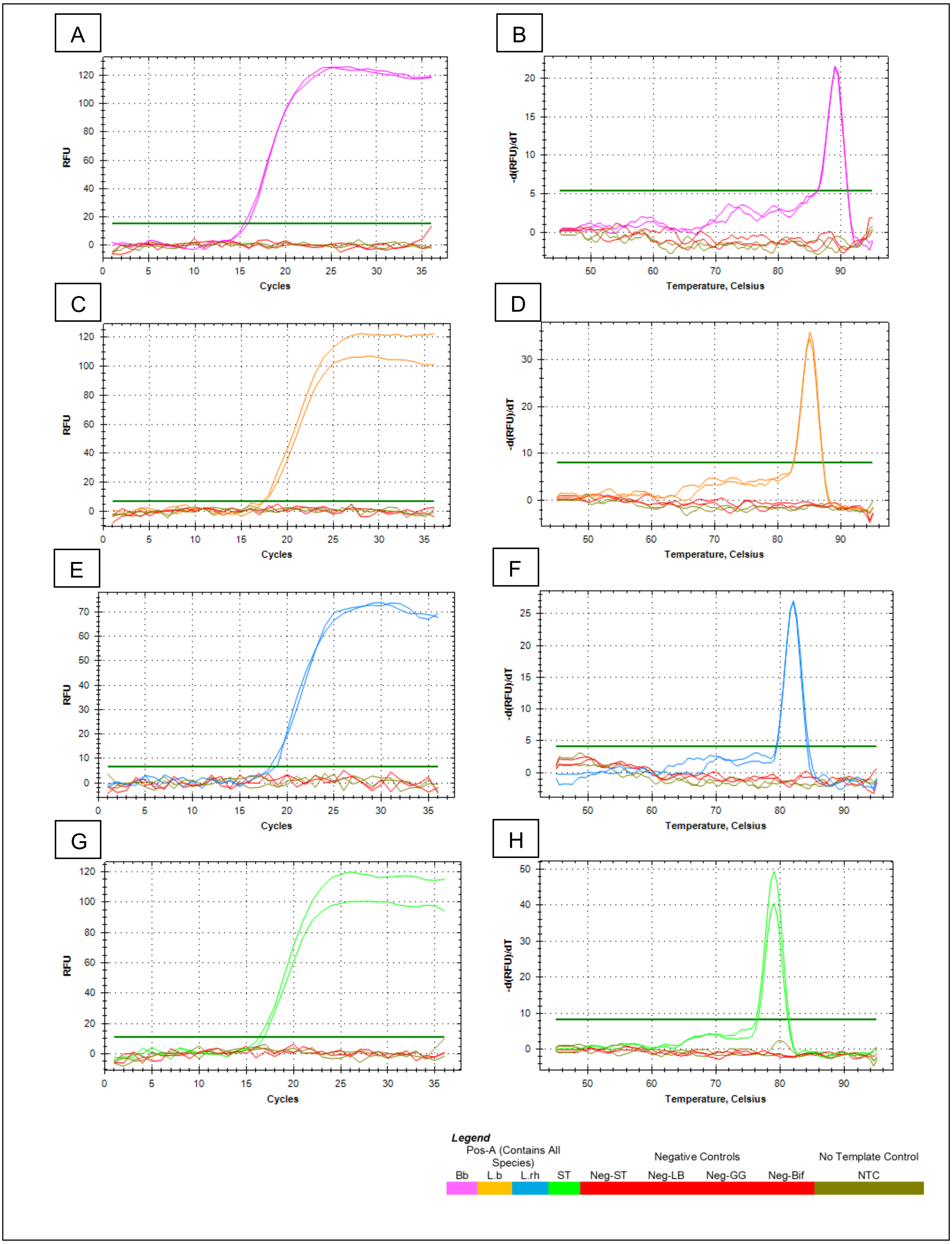

Figure S2. Amplification (left) and melt (right) curves showing the specificity of the four sets of primers in mixed species environment. Graphs A-B represented primers for B. bifidum (Bb), C-D for L. delbrueckii subsp. bulgaricus (L.b), E-F for L. rhamnosus (L.rh), and G-H for S. thermophilus (ST).

Standard Curves: Limit of Quantification (LOQ)

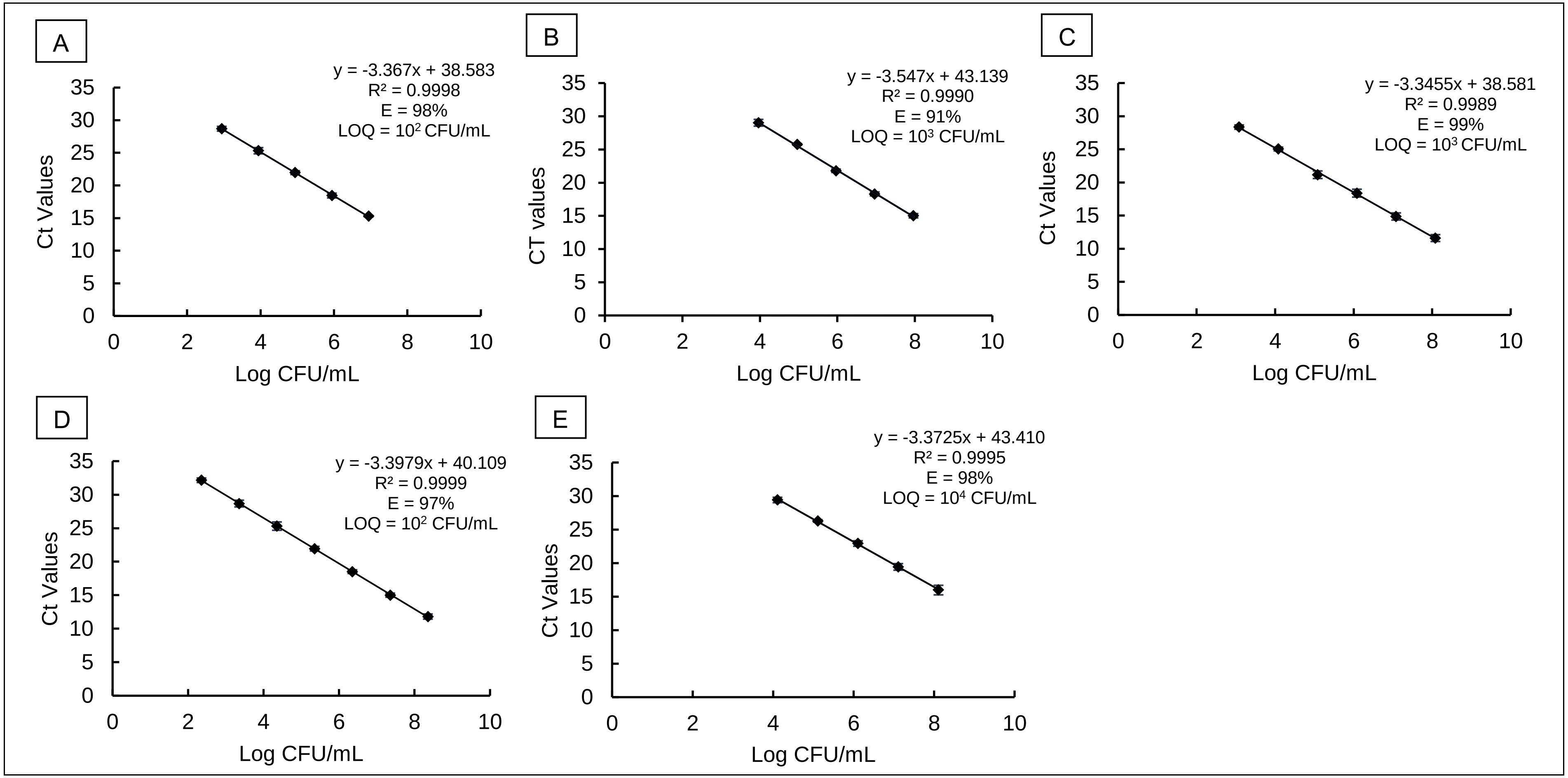

Figure S3. Standard curves of PMAxx™-qPCR assay created and used for determining the limit of quantification (LOQ) for *B. bifidum* ATCC 11863 (A), *B. breve* ATCC 15700 (B), *L. delbrueckii* subsp. *bulgaricus* (C), *S. thermophilus* (D), and *L. rhamnosus* (E). Each point represents the mean ± standard deviation of CT values of two independent runs (*n* = 4). Each run was carried out in duplicate
